# Angiotensin II–Stimulating Antihypertensive Medications and Dementia-Related Neuropathology

**DOI:** 10.1001/jamanetworkopen.2025.59113

**Published:** 2026-02-11

**Authors:** Shelly L. Gray, Onchee Yu, Nicole M. Gatto, Zachary A. Marcum, Caitlin S. Latimer, Nadia Postupna, Yu-Ru Su, Douglas Barthold, Jan Willem van Dalen, Edo Richard, C. Dirk Keene, Pamela A. Shaw, Linda K. McEvoy, Eric B. Larson, Paul K. Crane

**Affiliations:** 1 Department of Pharmacy, School of Pharmacy, University of Washington, Seattle; 2 Kaiser Permanente Washington Health Research Institute, Seattle, Washington; 3 Department of Laboratory Medicine and Pathology, University of Washington, Seattle; 4 Department of Public and Occupational Health, Amsterdam UMC, University of Amsterdam, Amsterdam, the Netherlands; 5 Department of Neurology, Donders Institute for Brain, Behaviour and Cognition, Radboud University Medical Centre, Nijmegen, the Netherlands; 6 School of Medicine, University of Washington, Seattle

## Abstract

**Importance:**

Antihypertensive medications that stimulate angiotensin II type 2 or 4 receptors (angiotensin II–stimulating medications) may be associated with lower risk of dementia.

**Objective:**

To examine associations between cumulative exposure to angiotensin II–stimulating vs angiotensin II–inhibiting antihypertensive medications and neuropathology, accounting for blood pressure.

**Design, Setting, and Participants:**

This community-based autopsy cohort study from the Adult Changes in Thought cohort was conducted at Kaiser Permanente Washington between February 24, 1994, and November 25, 2022, among 756 participants who had blood pressure measurements and at least 1 person-year (PY) of angiotensin II–stimulating or –inhibiting antihypertensive medication exposure prior to death. Statistical analysis was performed between September 2024 and August 2025.

**Exposure:**

Angiotensin II–stimulating antihypertensive medications (angiotensin II receptor blockers, dihydropyridine calcium channel blockers, thiazides) and angiotensin II–inhibiting antihypertensive medications (angiotensin-converting enzyme inhibitors, β-blockers, nondihydropyridine calcium channel blockers) were ascertained from paper-based medical records (before 1977) and electronic prescription fill data (after 1977). The primary exposure was cumulative angiotensin II PYs, and the secondary exposure was long-term use (≥15 years).

**Main Outcomes and Measures:**

Neuropathology outcomes were classified as Alzheimer disease related, vascular brain injury, or other. Exploratory outcomes included quantitative measures of Aβ42 and phosphorylated tau. Data were analyzed using multivariable modified Poisson, proportional odds, and linear regression models and accounted for potential selection bias.

**Results:**

The sample included 756 participants (mean [SD] age at death, 89.2 [6.4] years; 440 women [58.2%]; mean [SD] follow-up, 22.2 [13.5] years). Compared with exposure to 5 additional PYs of angiotensin II–inhibiting antihypertensive medications, exposure to 5 additional PYs of angiotensin II–stimulating antihypertensive medications was associated with a 6% lower risk for arteriolosclerosis (relative risk [RR], 0.94; 95% CI, 0.89-0.99), with long-term use associated with a 24% lower risk (RR, 0.76; 95% CI, 0.63-0.91). For exploratory outcomes, PYs of angiotensin II–stimulating antihypertensive medications were associated with less quantitative phosphorylated tau burden in several brain regions (temporal lobe [adjusted ratio of geometric means, 0.79; 95% CI, 0.62-1.00], hippocampus [adjusted ratio of geometric means, 0.83; 95% CI, 0.71-0.97], cornu ammonis subfield 1 [adjusted ratio of geometric means, 0.86; 95% CI, 0.74-0.99], and transentorhinal cortex [adjusted ratio of geometric means, 0.83; 95% CI, 0.70-0.98]) but not with Aβ42 quantitative measures.

**Conclusions and Relevance:**

In this community-based autopsy cohort study, angiotensin II–stimulating antihypertensive medications were associated with lower risk of neuropathological burden, supporting findings from epidemiologic dementia studies. Additional mechanistic research examining the effects of individual antihypertensive classes on Alzheimer disease–related biomarkers is warranted.

## Key Points

**Question:**

Are antihypertensive medications that stimulate vs inhibit angiotensin II type 2 or 4 receptors associated with lower risk for neuropathology burden beyond blood pressure control?

**Findings:**

In this cohort study with 756 decedents, cumulative angiotensin II–stimulating exposure was associated with lower risk for some types of neuropathology relative to angiotensin II–inhibiting exposure. Long-term angiotensin II–stimulating exposure (≥15 years) was associated with a 24% lower risk for arteriolosclerosis.

**Meaning:**

This study suggests that, relative to angiotensin II–inhibiting antihypertensive exposure, angiotensin II–stimulating antihypertensive medications were associated with lower risk of certain dementia-related neuropathologies.

## Teaser

This cohort study examines associations between cumulative exposure to angiotensin II–stimulating vs angiotensin II–inhibiting antihypertensive medications and neuropathology beyond blood pressure control.

## Introduction

Hypertension is a leading modifiable risk factor for dementia and affects nearly half of US adults aged 18 years or older.^1,2^ Although lower blood pressure (BP), especially in midlife, is associated with lower risk of dementia,^1^ it remains unclear whether particular classes of antihypertensive medications are more effective at reducing dementia risk, independent of their BP-lowering effects. Prevention is critical to reducing the public health burden of dementia, for example, through optimal treatment of modifiable factors such as hypertension.^1,3^

One approach for examining antihypertensive effects is to categorize these drugs according to their activity at angiotensin II receptors type 2 and 4. Those that result in more activity at these receptors (hereafter, *angiotensin II stimulating*) include angiotensin II receptor blockers (ARBs), dihydropyridine calcium channel blockers (CCBs), and thiazide diuretics. Those that result in less activity at these receptors (hereafter, *angiotensin II inhibiting*) include angiotensin-converting enzyme (ACE) inhibitors, β-blockers, and nondihydropyridine CCBs.^4^ Animal and in vitro data support the angiotensin hypothesis that angiotensin II–stimulating antihypertensive medications have beneficial effects on the brain, possibly through multiple pathways (eFigure 1 in Supplement 1).^5,6,7,8,9,10^ Although individual epidemiologic studies of the angiotensin hypothesis related to dementia outcomes have reported mixed findings,^4,11,12,13^ a 2025 systematic review and meta-analysis involving nearly 1.9 million people with hypertension reported that, compared with angiotensin II–inhibiting antihypertensive medications, angiotensin II–stimulating antihypertensive medications were associated with 13% lower risk of all-cause dementia and 12% lower risk of Alzheimer disease (AD) dementia.^14^

The mechanisms underlying these observations remain unclear. Previous autopsy studies have not examined the angiotensin hypothesis and have been limited by variable classifications of antihypertensive medications,^15,16,17,18,19^ crude medication exposure definitions (ie, ever vs never),^15,16,17,18,19,20,21,22^ and failure to adjust for BP.^15,16,17,20,21,22^ To this end, we examined associations between cumulative exposure to angiotensin II–stimulating vs –inhibiting antihypertensive medications and neuropathology among a community-based autopsy cohort study of older adults, while adjusting for longitudinal BP.

## Methods

This study was approved by the University of Washington Human Subjects Division and the Kaiser Permanente Interregional institutional review boards. Participants provided written informed consent. This report follows the Strengthening the Reporting of Observational Studies in Epidemiology (STROBE) reporting guideline.

### Overview and Data Sources

Adult Changes in Thought (ACT) is an ongoing prospective cohort study of older community-dwelling adults that began February 24, 1994, with an overall goal of understanding dementia risk factors. ACT enrolls members of Kaiser Permanente Washington (KPWA), an integrated health care delivery system, who are 65 years of age or older, have been KPWA members for 2 years or more at enrollment, and receive primary care at a greater Seattle area clinic. ACT methods have been described.^23^ In brief, participants confirmed to be dementia free at enrollment are assessed at study entry and biennially to evaluate cognitive function and are followed up with until dementia onset, death, or study discontinuation. Participants were enrolled in 3 waves: the original cohort (1994-1996), the expansion cohort (2000-2003), and continuous enrollment (≥2004). ACT collects information about demographic characteristics, medical history, health behaviors, and functional measures at study visits; obtains a blood sample for *APOE* genotyping; and has access to KPWA patient records from pharmacy dispensing data, paper-based medical records, and electronic health records, with the latter starting in the early 2000s.^23^

### Study Population

**Figure 1.**
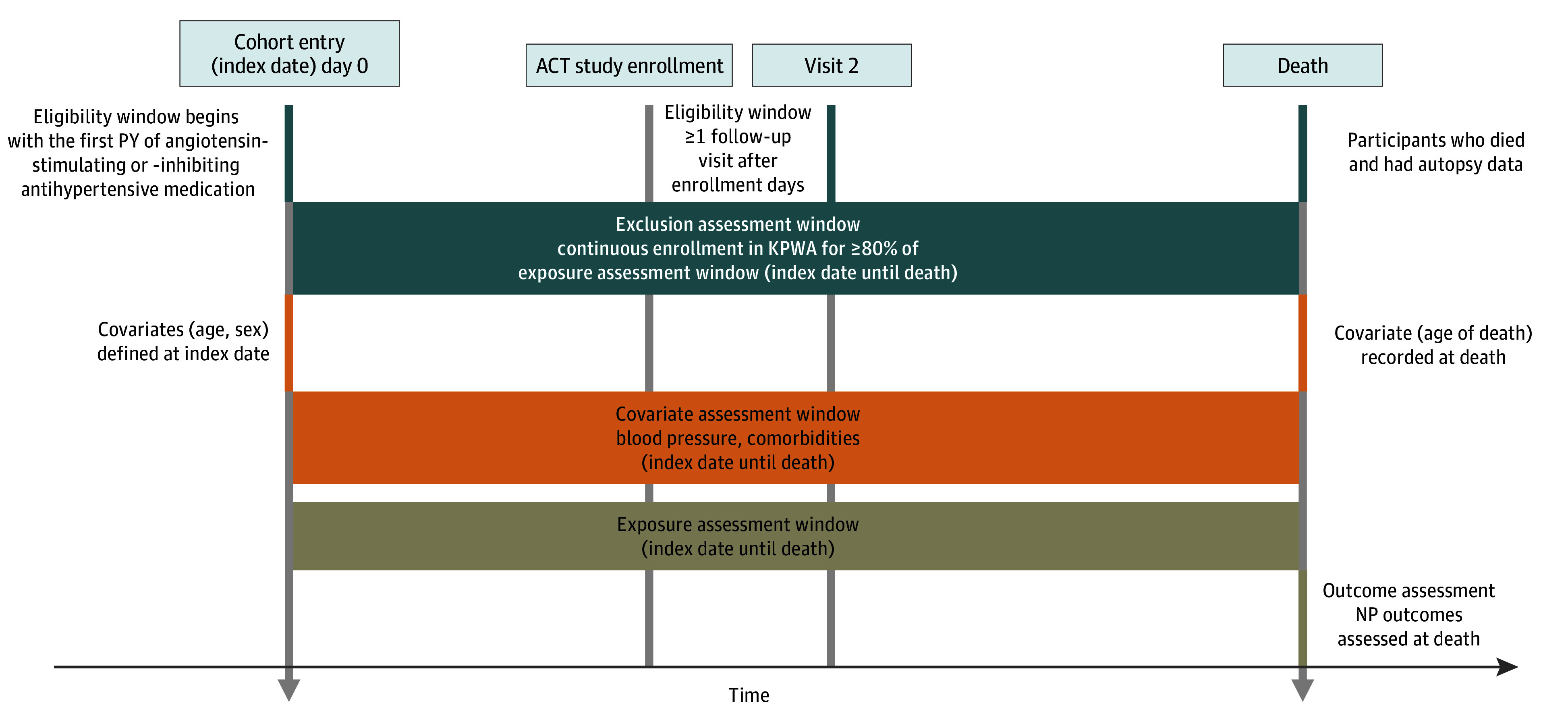
Study Design We defined the index date for a participant as their first year with a prescription fill for an angiotensin II antihypertensive medication (which could have occurred prior to enrollment in the Adult Changes in Thought [ACT] study). Eligible participants had at least 1 follow-up visit, consented to autopsy, and had neuropathology data and longitudinal blood pressure readings. KPWA indicates Kaiser Permanente Washington; NP, neuropathology; and PY, patient-year.

Eligible participants were enrolled in ACT as of November 25, 2022, had 1 person-year (PY) or more of angiotensin II antihypertensive exposure, had 1 biennial follow-up visit or more, and had neuropathology and BP data available (eFigure 2 in Supplement 1). Participants were followed up from the first recorded angiotensin II antihypertensive fill (index) date until death, and only those with continuous KPWA health plan enrollment for at least 80% of that period were included. The study design is illustrated in Figure 1.

### Antihypertensive Medication Exposure

Antihypertensive medication exposure was ascertained through manual review and abstraction of paper-based medical records that capture the year and name of each antihypertensive medication used before 1977^24^ and through prescription fills recorded in KPWA automated pharmacy dispensing data since 1977, which include drug name, strength, route of administration, and date and amount dispensed. Antihypertensive medications were classified as (1) angiotensin II stimulating (ie, ARBs, dihydropyridine CCBs, and thiazide diuretics), (2) angiotensin II inhibiting (ie, ACE inhibitors, β-blockers, and nondihydropyridine CCBs), and (3) all others (eTable 1 in Supplement 1).

The exposure measures of interest were cumulative years of angiotensin II–stimulating and –inhibiting antihypertensive medications, which were defined as the number of years of antihypertensive medication exposure in each category (separately) accumulating all years prior to death. A calendar year of exposure was defined as documentation of an angiotensin II–stimulating or –inhibiting antihypertensive medication in the paper-based medical record or at least 2 prescription fills from pharmacy dispensing data. When there was only 1 prescription fill for a medication in a year, 0.25 years of exposure or the equivalent of a 90-day of medication supply was assumed. Participants could switch between angiotensin II–stimulating and –inhibiting antihypertensive medications over time and thus contribute exposure time to both regimen types.

### Neuropathology

Neuropathology outcomes were assessed by board-certified neuropathologists (C.S.L. and C.D.K.) according to published guidelines.^25,26,27,28,29,30,31,32^ Most autopsies were performed less than 48 hours post mortem, with 40% within 8 hours. Brains, once removed, were fixed in 10% neutral buffered formalin for at least 14 days.

We examined AD neuropathology, vascular brain injury, and other neuropathology. We defined high levels of pathology for each outcome based on established cutpoints in the literature (eTable 2 in Supplement 1). AD neuropathology included cortical neuritic plaque density (Consortium to Establish a Registry for Alzheimer Disease [CERAD] level),^33^ neurofibrillary tangle distribution (Braak stage),^34,35^ Aβ plaque distribution (Thal phase),^36^ and presence of cerebral amyloid angiopathy.^37^ The AD neuropathologic change (ADNC) score is a composite measure based on Thal phase, Braak stage, and CERAD level.^25^

Vascular brain injury parameters included the presence of cerebral microinfarcts and macroscopic infarcts as well as the severity of atherosclerosis and arteriolosclerosis.^38^ Other outcomes included the distribution of Lewy bodies by brain region^38^ and the presence of limbic-predominant age-related TDP-43 (transactive response DNA-binding protein 43) encephalopathy (LATE).^39^ For Lewy body disease, we focused on neocortical involvement, which is more consistently associated with cognitive impairment than those in other regions.^40^

Exploratory outcomes included quantitative measures of Aβ42 and phosphorylated tau available for a subset of the sample. Aβ42 in the frontal, occipital, temporal, and parietal regions was measured by Luminex assay (Luminex Corp) in extracts from formalin-fixed paraffin-embedded tissue.^32^ Phosphorylated tau burden was measured in 5-µm formalin-fixed paraffin-embedded sections labeled with anti-phospho–tau antibody (clone AT8; Thermofisher, MN1020), using HALO digital image analysis software (Indica Labs) in the frontal, occipital, temporal, and parietal cortex and the hippocampal regions (subfields CA1, CA2, CA3, CA4, dentate gyrus, and subiculum). To align with areas measured for Braak stages I to IV, we examined CA1, the entorhinal cortex, subiculum, and transentorhinal cortex, separately.^32^

### Covariates

Data for potential confounders selected from previous literature^4,11,12^ were obtained from ACT study visits, electronic health records, and manual abstraction of paper-based medical records (eTable 3 in Supplement 1). Demographic characteristics included age at death, age at first known angiotensin II antihypertensive medication use, sex, and self-reported race and ethnicity. The race and ethnicity categories included American Indian or Alaska Native, Asian, Black, Hispanic, Native Hawaiian or Pacific Islander, White, or other race and ethnicity (multiple races and ethnicities). American Indian or Alaska Native race and Hispanic ethnicity were condensed into the other race and ethnicity group to avoid cells with small counts. Race and ethnicity data were collected to describe the study sample. Additional confounders included systolic and diastolic BP as well as history of atrial fibrillation, diabetes, myocardial infarction, stroke, and heart failure during follow-up, as these medical conditions guide antihypertensive medication selection.

### Statistical Analysis

Statistical analysis was performed between September 2024 and August 2025. We modeled the exposure of cumulative years of angiotensin II–stimulating and –inhibiting antihypertensive medications in 2 ways: total PYs of use (primary, continuous exposure) and long-term use (secondary, binary exposure), defined as 15 years or more of cumulative exposure, which reflected the top 25% of angiotensin II–stimulating antihypertensive medication exposure in our sample. To examine associations, we used multivariable modified Poisson regression for binary neuropathologic outcomes to estimate relative risks (RRs) directly (because outcomes were not rare); ordinal logistic regression assuming proportional odds to analyze the presence of Lewy bodies in the neocortical region vs limbic only, brainstem only, or absence of Lewy bodies; and linear models for the quantitative exploratory outcomes. We report associated difference in outcomes (eg, mean difference for continuous outcomes, RR for binary outcomes, and odds ratio for ordinal outcomes) for 5 additional PYs of angiotensin II–stimulating antihypertensive medication relative to 5 additional PYs of angiotensin II–inhibiting antihypertensive medication exposure. We also compared long-term users (≥15 years) of angiotensin II–stimulating medications with long-term users of angiotensin II–inhibiting antihypertensive medications. For the exploratory continuous outcomes, due to skewness from a few very large values, we modeled log-transformed outcomes in linear regressions and estimated the fold change in the geometric means comparing 5 additional PYs of angiotensin II–stimulating antihypertensive medication exposure relative to 5 additional PYs of angiotensin II–inhibiting antihypertensive medication exposure.

Models adjusted for ACT study cohort; age at death; age at first known angiotensin II antihypertensive medication use; sex; history of atrial fibrillation, diabetes, myocardial infarction, stroke, and heart failure during follow-up; mean of annual mean diastolic and systolic BP in the years with angiotensin II antihypertensive medication use; and other antihypertensive medication exposure. Age and BP were modeled as natural cubic splines with 2 knots at the one-third and two-third percentiles to allow flexible adjustments for their associations with the outcomes to control for confounding. We applied a 2-sided α of .05, without multiple comparison correction because many neuropathological outcomes that we evaluated are not well studied, and we did not want to miss potential associations. Found associations are interpreted as hypothesis generating. We used generalized raking,^41^ which is equivalent to augmented inverse probability weighting,^42^ to efficiently account for selection into the autopsy sample (eMethods in Supplement 1).

In sensitivity analyses, we modeled uncontrolled BP as the proportion of years with annual mean systolic BP of 140 mm Hg or higher or diastolic BP of 90 mm Hg or higher. We ran models unadjusted for BP to examine the extent of confounding associated with this covariate. To examine associations in the group with a known start date of antihypertensive therapy, we restricted to new users of antihypertensive medications, defined as those having no antihypertensive medication use in the year prior to index (n = 625).

## Results

The total sample included 756 participants (mean [SD] age at death, 89.2 [6.4] years; mean [SD] age at index, 67.0 [14.1] years; 440 women [58.2%] and 316 men [41.8%]) (Table). The sample included 16 Asian individuals (2.1%), 6 Black individuals (0.8%), 710 White individuals (93.9%), and 24 individuals who identified as other race or ethnicity. By time of death, about one-third of participants had a history of diabetes, and over one-half had a history of stroke, myocardial infarction, atrial fibrillation, coronary artery disease, or heart failure. Characteristics varied according to angiotensin II antihypertensive medication exposure type and duration (<15 years, ≥15 years) (eTable 4 in Supplement 1).

**Table.** Characteristics of Antihypertensive Users in ACT Study Autopsy Sample

Characteristic	No. (%) (N = 756)
**ACT study entry**	
ACT study cohort	
Original	428 (56.6)
Expansion	171 (22.6)
Replacement	157 (20.8)
Age at index, mean (SD), y	67.0 (14.1)
Age at death, mean (SD), y	89.2 (6.4)
Time from first angiotensin II antihypertensive medication to death, mean (SD), y	22.2 (13.5)
Sex	
Female	440 (58.2)
Male	316 (41.8)
Race and ethnicity^a^^,^^b^	
Asian	16 (2.1)
Black	6 (0.8)
Other	24 (3.0)
White	710 (93.9)
Some college education^b^	551 (72.9)
Fair to poor self-rated health^b^	200 (26.5)
Regular exercise^b^^,^^c^	283 (37.4)
*APOE* ε4^b^	203 (26.9)
**Summarized over study follow-up (at death)**	
Blood pressure	
Annual systolic blood pressure, mean (SD), mm Hg^d^	135.8 (12.6)
Annual diastolic blood pressure, mean (SD), mm Hg^d^	73.8 (7.1)
No. of years with uncontrolled blood pressure, mean (SD)^e^	6.7 (6.9)
Antihypertensive medication	
Angiotensin II–stimulating antihypertensive medication	
Ever used	584 (77.2)
Total No. of person-years of use, mean (SD)	9.3 (10.0)
Angiotensin II–inhibiting antihypertensive medication	
Ever used	706 (93.4)
Total No. of person-years of use, mean (SD)	12.2 (9.2)
Type of angiotensin II antihypertensive medication ever used	
Stimulating only	50 (6.6)
Inhibiting only	172 (22.8)
Both types	534 (70.6)
Any use of other antihypertensive medication	551 (72.9)
History of comorbidities at death	
Diabetes	245 (32.4)
Stroke	429 (56.7)
Myocardial infarction	417 (55.2)
Atrial fibrillation	429 (56.7)
Heart failure	507 (67.1)
Coronary artery disease	456 (60.3)
High depressive symptoms^f^	463 (61.2)
Dementia^g^	354 (46.8)
Alzheimer disease^g^	292 (38.6)

Abbreviation: ACT, Adult Changes in Thought.

^a^ Other race and ethnicity included American Indian or Alaska Native, Hispanic ethnicity, and other races or multiple races.

^b^ Missing data for race, 1 (0.1%); education, 7 (0.9%); self-rated health, 87 (11.5%); exercise, 82 (10.8%); and *APOE*, 25 (3.3%). All reported percentages in table are among participants with available data.

^c^ Performing 1 of several listed activities for 15 minutes or more, 3 times or more per week.

^d^ Mean annual systolic and diastolic blood pressure defined as each individual’s mean of annual systolic and diastolic blood pressure across the years with angiotensin II antihypertensive medication exposure.

^e^ Uncontrolled blood pressure defined as systolic blood pressure of 140 mm Hg or higher and/or diastolic blood pressure of 90 mm Hg or higher.

^f^ Center for Epidemiologic Studies Depression scale of 10 or more or *International Classification of Diseases* diagnosis.

^g^ Participants who screened positive for cognitive impairment during an ACT study visit underwent a standardized diagnostic evaluation for dementia by a multidisciplinary consensus conference and standard criteria were used for diagnoses of dementia and Alzheimer disease.

The mean (SD) time from first recorded antihypertensive medication use to death was 22.2 (13.5) years (Table). Most participants (584 [77.2%]) had exposure to angiotensin II–simulating antihypertensive medications, with a mean (SD) of 9.3 (10.0) PYs. An even higher proportion (706 [93.4%]) had exposure to an angiotensin II–inhibiting antihypertensive medication, with a mean (SD) of 12.2 (9.2) PYs. Most participants (534 [70.6%]) used both regimen types, with 172 (22.8%) using exclusively angiotensin II–inhibiting antihypertensive medications and 50 (6.6%) using exclusively angiotensin II–stimulating antihypertensive medications. Thiazides (68.4% [5417.0 of 7915.8 PYs]) accounted for the highest proportion of PYs for stimulating regimens and β-blockers (53.9% [6199.8 of 11 496.9 PYs]) accounted for the highest proportion of PYs for inhibiting regimens; 19.0% (2183.8 of 11 496.9 PYs) was β-blocker monotherapy (eTable 5 in Supplement 1). See eFigure 3 and eFigure 4 in Supplement 1 for further details about exposure patterns.

**Figure 2.**
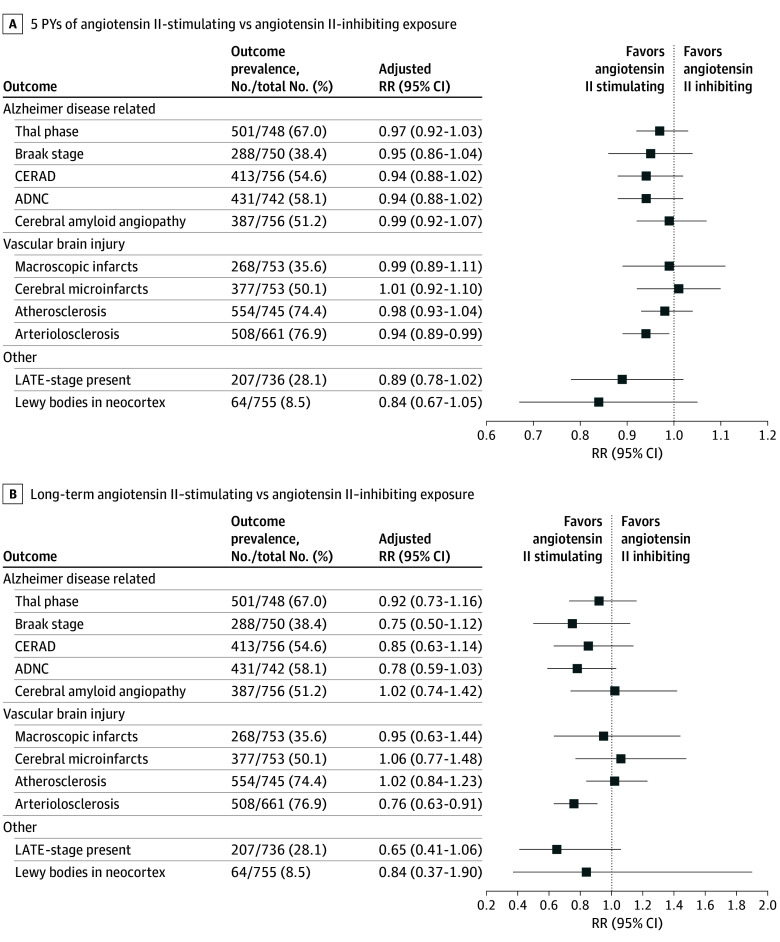
Associations Between Cumulative Person-Years (PYs) and Long-Term Antihypertensive Exposures and Neuropathology Outcomes A, Relative risk (RR) estimated from modified Poisson regression for each neuropathology outcome comparing 5 additional PYs of angiotensin II–stimulating with 5 additional PYs of angiotensin II–inhibiting antihypertensive medication exposure. B, RR estimated from modified Poisson regression for each neuropathology outcome comparing long-term exposure (≥15 years) to angiotensin II–stimulating antihypertensive medications with long-term exposure to angiotensin II–inhibiting antihypertensive medications. High level of neuropathology, defined as Thal phase (3-5), Braak stage (V or VI), Consortium to Establish a Registry for Alzheimer Disease (CERAD) level (moderate or frequent), Alzheimer disease neuropathologic change (ADNC) score (intermediate or high), cerebral amyloid angiopathy (any), macroscopic infarcts (any), cerebral infarcts (any), atherosclerosis (moderate or severe), arteriolosclerosis (moderate or severe), and limbic-predominant age-related TDP-43 (transactive response DNA-binding protein 43) encephalopathy (LATE) present (2-3). Models for macroscopic infarcts did not adjust for stroke because of collinearity expected between these 2 variables. Lewy bodies in neocortex results are presented as odds ratios from ordinal logistic regression (the risk of presence of Lewy bodies in the neocortical region vs in the limbic or brainstem regions or absence of Lewy bodies). Models adjusted for age at death, age at first known antihypertensive medication use, sex, Adult Changes in Thought cohort, mean annual diastolic and systolic blood pressure across the years with angiotensin II antihypertensive medication exposure, use of other antihypertensive medications, and history of atrial fibrillation, diabetes, myocardial infarction, stroke, and heart failure any time prior to death.

The distribution of the neuropathology outcomes is provided in eTable 6 in Supplement 1. High levels of neuropathology occurred in more than 50% of participants for Thal phase, CERAD level, ADNC score, cerebral amyloid angiopathy, cerebral microinfarcts, atherosclerosis, and arteriolosclerosis (Figure 2). Select characteristics according to neuropathology outcomes are provided in eTable 7 in Supplement 1.

### AD Neuropathology, Vascular Brain Injury, and Other Outcomes

Compared with exposure to 5 additional PYs of angiotensin II–inhibiting antihypertensive medications, exposure to 5 additional PYs of angiotensin II–stimulating antihypertensive medications was associated with a 6% lower risk of arteriolosclerosis (RR, 0.94; 95% CI, 0.89-0.99) (Figure 2A). Long-term use of angiotensin II–simulating compared with angiotensin II–inhibiting antihypertensive medications was associated with a 24% lower risk of arteriolosclerosis (RR, 0.76; 95% CI, 0.63-0.91) (Figure 2B).

Five additional PYs of angiotensin II–stimulating antihypertensive medications was associated with a nonstatistically significantly lower risk, by 5% to 6%, for AD neuropathology (Braak stage, CERAD level, ADNC score), an 11% lower risk of LATE-stage pathology present, and 16% lower risk for Lewy bodies in the neocortex, compared with exposure to 5 additional PYs of angiotensin II–inhibiting antihypertensive medications (Figure 2A). In sensitivity analyses, estimates were similar between models with and models without BP adjustment (eFigure 5 in Supplement 1), when BP was quantified as the proportion of years with uncontrolled BP (eFigure 6 in Supplement 1) and when restricting to participants with new use of antihypertensive medications (eFigure 7 in Supplement 1).

### Exploratory Outcomes

**Figure 3.**
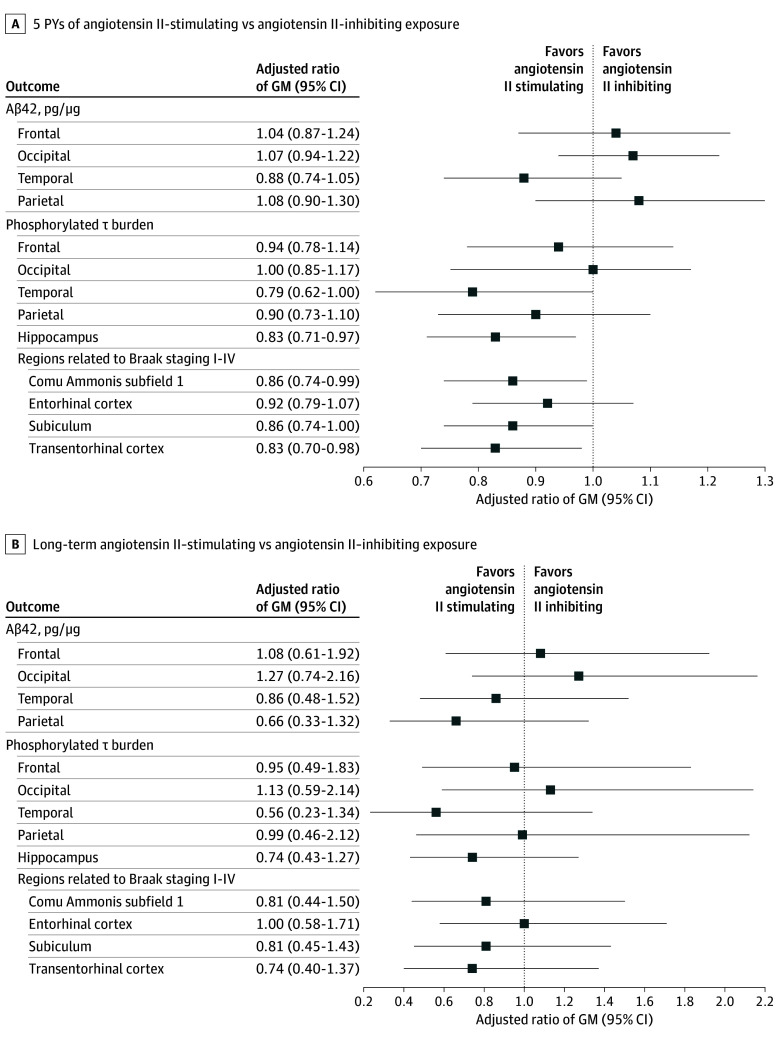
Associations Between Cumulative Person-Years (PYs) and Long-Term Antihypertensive Exposures and Exploratory Neuropathology Outcomes A, The ratio of geometric means (GMs) of each neuropathology outcome comparing 5 additional PYs of angiotensin II–stimulating with 5 additional PYs of angiotensin II–inhibiting antihypertensive medication exposure. B, The ratio of GMs of each neuropathology outcome comparing long-term exposure (≥15 years) to angiotensin II–stimulating antihypertensive medications with long-term exposure to angiotensin II–inhibiting antihypertensive medications. Hippocampus subregions included CA1, CA2, CA3, CA4, dentate gyrus, and subiculum. Linear model adjusted for age at death, age at first known antihypertensive medication use, sex, Adult Changes in Thought cohort, mean annual diastolic and systolic blood pressure across the years with angiotensin II antihypertensive medication exposure, use of other antihypertensive medications, and history of atrial fibrillation, diabetes, myocardial infarction, stroke, and heart failure any time prior to death. Age and blood pressure were modeled as natural cubic splines with 2 knots at the tertile.

There were no differences between angiotensin II regimen types and measures of Aβ42 (Figure 3A and B). Five additional PYs of angiotensin II–stimulating vs angiotensin II–inhibiting antihypertensive medications was associated with 21% less hyperphosphorylated tau burden in the temporal lobe (adjusted ratio of geometric means, 0.79; 95% CI, 0.62-1.00), 17% less in the hippocampus (adjusted ratio of geometric means, 0.83; 95% CI, 0.71-0.97), 14% less in the CA1 subfield (adjusted ratio of geometric means, 0.86; 95% CI, 0.74-0.99), and 17% less in the transentorhinal cortex (adjusted ratio of geometric means, 0.83; 95% CI, 0.70-0.98) (Figure 3A). Long-term use was not associated with phosphorylated tau burden for these regions (Figure 3B).

## Discussion

We present the first investigation, to our knowledge, of angiotensin II–stimulating compared with angiotensin II–inhibiting antihypertensive medications in a community-based autopsy cohort with a broad range of neuropathological outcomes, including quantitative measures of Aβ42 and total phosphorylated tau, accounting for longitudinal BP control. Exposure to angiotensin II–stimulating antihypertensive medications was associated with lower levels of arteriolosclerosis, which was more pronounced among individuals with 15 years or more of exposure, with an RR reduction of 24%. This finding was consistent across multiple sensitivity analyses. Angiotensin II–stimulating regimens were associated with lower levels of phosphorylated tau in several brain regions, ranging from 14% to 21%, but not with quantitative Aβ42 measures. Although not statistically significant, exposure to angiotensin II–stimulating antihypertensive medications was associated with lower levels of several standard neuropathology measures (Braak stage, CERAD level, ADNC score, LATE-stage pathology, and Lewy bodies in the neocortex), ranging from 5% to 16% with the primary exposure.

Our findings are not directly comparable with previous autopsy studies, which did not categorize antihypertensive medications based on angiotensin II activity, and either aggregated all antihypertensive medications together,^15,16,17,20,21,22^ combined medications acting via the renin-angiotensin system (ie, ARBs and ACE inhibitors together),^19^ or compared ARBs vs other antihypertensive medications.^18^ Furthermore, we focused on quantifying PYs of exposure compared with other studies’ binary indicator of ever or never use.

We did not find significant differences in Aβ outcomes between regimen types, as might be expected from prior studies comparing ARBs and ACE inhibitors, examples of angiotensin II–stimulating and –inhibiting medications, respectively.^18,43,44^ Research supporting the angiotensin hypothesis has centered in part on the finding that ACE is associated with enzymatic Aβ degradation, suggesting ACE inhibitors may impair Aβ clearance, whereas ARBs preserve ACE function. Studies have found less amyloid burden for ARB treatment compared with ACE inhibitor treatment.^18,43,44^ A subanalysis of 362 participants in a neuropathologic study of 890 participants with cognitive impairment and hypertension showed significantly lower amyloid and neuritic plaque burden in ARB-treated participants compared with ACE inhibitor users.^18^ An in vivo positron emission tomographic imaging study of 311 participants (142 with normal cognition and 169 with Aβ-positive AD or mild cognitive impairment) revealed slower cortical Aβ accumulation over 48 months among adults with normal cognition receiving ARBs vs ACE inhibitors but no difference in rate of accumulation among those with AD or mild cognitive impairment.^43^ Although our study is not directly comparable with these studies, our findings implicate pathologic mechanisms other than Aβ in potential protective associations of angiotensin II–stimulating exposure.

β-Blockers accounted for 53.9% of angiotensin II–inhibiting exposure in this study, and 19% was β-blocker monotherapy. Since the early 2000s, hypertension guidelines have increasingly deemphasized β-blockers as first-line therapy, due partly to inferior stroke prevention compared with other antihypertensive classes.^45^ This lower effectiveness in stroke prevention appears independent of any angiotensin-related mechanisms. If β-blocker exposure was associated with poorer stroke prevention and increased vascular pathology in this sample, this could have biased results in favor of angiotensin II–stimulating antihypertensive medications, potentially exaggerating their apparent superiority in reducing risk of vascular injury parameters. However, most vascular injury outcomes did not differ by treatment regimen, suggesting this bias had limited impact.

### Strengths and Limitations

Our study has some strengths. A major strength is the extensive longitudinal BP data (mean follow-up, 22 years) allowing adjustments throughout the exposure period, whereas prior neuropathology studies did not adjust for BP or adjusted for BP over a short follow-up time (median, 2 years, or only at baseline).^18,19^ Nonetheless, it is difficult to adjust for BP given the complex association between BP and dementia risk that varies by age.^46,47^ Midlife hypertension has consistently been associated with higher risk of all-cause dementia and AD; however, in late life, this association has not been regularly observed.^1^ We used a flexible spline to model BP; however, this strategy does not account for this complex association.

Additional strengths include the community-based autopsy cohort and the robust data on decades-long medication exposures. We used rigorous statistical methods to address selection bias.

Our study also has some limitations. Although our sample size is large by autopsy standards, it yielded wide 95% CIs, and we cannot exclude meaningful associations. Moreover, the angiotensin hypothesis represents only 1 mechanistic framework, and other cellular pathways may underlie the observed associations. Evaluating associations among participants taking only a single angiotensin II regimen (“pure users”) or by antihypertensive medication subclass would offer additional insights, but our small sample did not provide the power to evaluate these subgroups. Our analytic approach reflects clinical practice, in which people commonly use multiple antihypertensive agents concurrently or over time. Evolving antihypertensive medication prescribing patterns over the follow-up period may limit generalizability to current clinical practice. Although we adjusted for numerous potential confounders, including chronic conditions that could influence preferential prescribing of antihypertensive medications, as with any observational study, unmeasured or residual confounding could confound our estimates. We did not correct for multiple comparisons; thus, results should be interpreted cautiously. The study population was predominantly White and well educated, which may limit generalizability.

## Conclusions

In this autopsy cohort study, angiotensin II–stimulating compared with angiotensin II–inhibiting antihypertensive medications were associated with a lower burden of key neuropathology. These observations were consistent with and extend the findings of prior observational studies that explored the angiotensin hypothesis in dementia. These findings should be confirmed by additional research. Given the advances made with in vivo imaging and cerebrospinal fluid–based and blood-based biomarkers, additional mechanistic research examining the effects of individual antihypertensive classes on AD-related biomarkers is warranted.

## Notes

Supplement 1. **eMethods.** Accounting for Selection Bias
eReferences.
**eTable 1.** Antihypertensive Medications (Generic Name) by Category **eTable 2.** Description of Outcomes **eTable 3.** Sources of Exposure and Covariate Data **eTable 4.** Characteristics of ACT Autopsy Sample By Long-Term Angiotensin II Antihypertensive Exposures **eTable 5.** Person-Years (PYs) of Exposure According to Antihypertensive Class **eTable 6.** Distribution of Neuropathology Outcomes **eTable 7.** Characteristics of ACT Autopsy Sample by Neuropathology Outcome Status **eFigure 1.** Activity of Antihypertensive Sub-Classes in the Renin-Angiotensin System **eFigure 2.** Flow Diagram of Study Sample **eFigure 3.** Person-Years (PYs) of Exposure by Calendar Year According to Type of Angiotensin II Exposure **eFigure 4.** Person-Years (PYs) of Exposure by Calendar Year According to Angiotensin II Antihypertensive Subclass **eFigure 5.** Associations Between Cumulative Person-Years (PYs) of Antihypertensive Exposure and Neuropathology Outcomes Not Adjusting for Blood Pressure **eFigure 6.** Associations Between Cumulative Person-Years (PYs) of Antihypertensive Exposure and Neuropathology Outcomes Adjusting for Uncontrolled Blood Pressure **eFigure 7.** Associations Between Cumulative Person-Years (PYs) of Antihypertensive Exposure and Neuropathology Outcomes in New Users of Antihypertensives

Supplement 2.
Data Sharing Statement
