# Supplementary material for: Angiotensin II–Stimulating Antihypertensive Medications and Dementia-Related Neuropathology
Source: JAMA Netw Open. 2026 Feb 11;9(2):e2559113. doi: 10.1001/jamanetworkopen.2025.59113 (PMC12895290; doi:10.1001/jamanetworkopen.2025.59113)
Supplement: Supplement 1. — eMethods. Accounting for Selection Bias eReferences. eTable 1. Antihypertensive Medications (Generic Name) by Category eTable 2. Description of Outcomes eTable 3. Sources of Exposure and Covariate Data eTable 4. Characteristics of ACT Autopsy Sample By Long-Term Angiotensin II Antihypertensive Exposures eTable 5. Person-Years (PYs) of Exposure According to Antihypertensive Class eTable 6. Distribution of Neuropathology Outcomes eTable 7. Characteristics of ACT Autopsy Sample by Neuropathology Outcome Status eFigure 1. Activity of Antihypertensive Sub-Classes in the Renin-Angiotensin System eFigure 2. Flow Diagram of Study Sample eFigure 3. Person-Years (PYs) of Exposure by Calendar Year According to Type of Angiotensin II Exposure eFigure 4. Person-Years (PYs) of Exposure by Calendar Year According to Angiotensin II Antihypertensive Subclass eFigure 5. Associations Between Cumulative Person-Years (PYs) of Antihypertensive Exposure and Neuropathology Outcomes Not Adjusting for Blood Pressure eFigure 6. Associations Between Cumulative Person-Years (PYs) of Antihypertensive Exposure and Neuropathology Outcomes Adjusting for Uncontrolled Blood Pressure eFigure 7. Associations Between Cumulative Person-Years (PYs) of Antihypertensive Exposure and Neuropathology Outcomes in New Users of Antihypertensives [file jamanetwopen-e2559113-s001.pdf]

Supplementary Online Content

Gray SL, Yu O, Gatto NM, et al. Angiotensin II–stimulating antihypertensive medications and dementia-related neuropathology. *JAMA Netw Open.* 2026;9(2):e2559113. doi:10.1001/jamanetworkopen.2025.59113

eMethods. Accounting for Selection Bias
eReferences.
eTable 1. Antihypertensive Medications (Generic Name) by Category
eTable 2. Description of Outcomes
eTable 3. Sources of Exposure and Covariate Data
eTable 4. Characteristics of ACT Autopsy Sample by Long-Term Angiotensin II Antihypertensive Exposures
eTable 5. Person-Years (PYs) of Exposure According to Antihypertensive Class
eTable 6. Distribution of Neuropathology Outcomes
eTable 7. Characteristics of ACT Autopsy Sample by Neuropathology Outcome Status
eFigure 1. Activity of Antihypertensive Sub-Classes in the Renin-Angiotensin System
eFigure 2. Flow Diagram of Study Sample
eFigure 3. Person-Years (PYs) of Exposure by Calendar Year According to Type of Angiotensin II Exposure
eFigure 4. Person-Years (PYs) of Exposure by Calendar Year According to Angiotensin II Antihypertensive Subclass
eFigure 5. Associations Between Cumulative Person-Years (PYs) of Antihypertensive Exposure and Neuropathology Outcomes Not Adjusting for Blood Pressure
eFigure 6. Associations Between Cumulative Person-Years (PYs) of Antihypertensive Exposure and Neuropathology Outcomes Adjusting for Uncontrolled Blood Pressure
eFigure 7. Associations Between Cumulative Person-Years (PYs) of Antihypertensive Exposure and Neuropathology Outcomes in New Users of Antihypertensives

This supplementary material has been provided by the authors to give readers additional information about their work.

© 2026 Gray SL et al. *JAMA Network Open.*

eMethods. Accounting for Selection Bias

Selection bias may occur if demographic and clinical factors are associated with inclusion in the autopsy cohort (including consent to autopsy, study withdrawal, and death).[1] We used generalized raking, equivalent to a type of augmented inverse probability weighting (AIPW, described in detail below), to account for differences that might exist between the analytic sample and the broader ACT cohort. First, Horvitz-Thompson weights were derived from the inverse of inclusion probability estimated from a logistic regression model of being included in the analysis of an outcome (i.e. had the specific neuropathological outcome, BP data and met enrollment requirement) using all 3970 ACT participants prescribed an angiotensin II stimulating or inhibiting antihypertensive with at least one biennial follow-up visit (Ang II cohort) (**eFigure 1**). Since not all participants had all the neuropathological outcomes, a separate selection model was performed for each outcome. Predictors in the selection model included age at last ACT visit, ACT study cohort, sex, any college education, fair or poor self-rated health, *APOE* genotype, dementia, history of stroke, coronary artery disease, atrial fibrillation, heart failure and diabetes diagnoses. All covariates were defined in **eTable 3** except that no chart review data were used since abstraction has not been completed for all participants without autopsy.

We then used generalized raking[2] to calibrate the inverse probability weighting (IPW) weights in order to incorporate information from participants in the Ang II cohort who lacked data for the neuropathology outcome. Following the procedures of Breslow et al[3] to improve the efficiency of the IPW estimator, we approximated the optimal raking variables by calibrating the IPW weights with the estimated influence functions for each regression coefficient in the target outcome model. Specifically, we used the variables from the selection and outcome models available on the Ang II cohort to impute the missing neuropath outcomes on the entire Ang II cohort. The target outcome model was then fit with the imputed outcome to generate estimates of efficient influence functions for each regression coefficient in the model. The average of these values across multiple imputations was used to calibrate the IPW weights.[4] Lumley et al. [5] showed that for the optimal calibration variable, this generalized raking procedure is asymptotically equivalent to the efficient AIPW estimator. Inference is conducted using a sandwich variance estimator. All analyses were conducted using the survey[6] package (version 4.4.2) in R (version 4.4.3).[7]

© 2026 Gray SL et al. *JAMA Network Open.*

eReferences.

1. Hamel E, Royea J, Ongali B, Tong XK. Neurovascular and Cognitive failure in Alzheimer's Disease: Benefits of Cardiovascular Therapy. *Cell Mol Neurobiol*. 2016;36(2):219-32. doi:10.1007/s10571-015-0285-4
2. Deville J-C, Särndal C-E, Sautory O. Generalized raking procedures in survey sampling. *J Am Stat Assoc*. 1993;88(423):1013-1020.
3. Breslow NE, Lumley T, Ballantyne CM, Chambless LE, Kulich M. Improved Horvitz-Thompson Estimation of Model Parameters from Two-phase Stratified Samples: Applications in Epidemiology. *Stat Biosci*. May 1 2009;1(1):32. doi:10.1007/s12561-009-9001-6
4. Han K, Shaw PA, Lumley T. Combining multiple imputation with raking of weights: An efficient and robust approach in the setting of nearly true models. *Stat Med*. Dec 30 2021;40(30):6777-6791. doi:10.1002/sim.9210
5. Lumley T, Shaw PA, Dai JY. Connections between survey calibration estimators and semiparametric models for incomplete data. *Int Stat Rev*. 2011;79(2):200-220. doi:10.1111/j.1751-5823.2011.00138.x
6. Lumley T, Gao P, Schneider B. Analysis of Complex Survey Samples. March 20. https://cran.r-project.org/web/packages/survey/survey.pdf
7. Team RC. R: A Language and Environment for Statistical Computing. R Foundation for Statistical Computing. https://www.r-project.org/

eTable 1. Antihypertensive Medications (Generic Name) by Category

<table>
<tr><th>Angiotensin II-stimulating</th><th>Angiotensin II-inhibiting</th><th>Other</th></tr>
<tr><td><u>Dihydropyridine calcium channel blockers</u>
amlodipine
amlodipine/celecoxib
felodipine
isradipine
levamlodipine
mibefradil
nicardipine
nifedipine
nimodipine
nisoldipine</td><td><u>Angiotensin-converting enzyme inhibitors</u>
benazepril
captopril
enalapril
fosinopril
lisinopril
moexipril
perindopril
quinapril
ramipril
trandolapril</td><td rowspan="3">acetazolamide
aliskiren
amiloride
bumetanide
clonidine
deserpidine
doxazosin
eplerenone
ethacrynic acid
furosemide
guanabenz
guanadrel
guanethidine
guanfacine
hydralazine
isosorbide
mannitol
mecamylamine
methyldopa
metolazone
Minoxidil
phenoxybenzamine
prazosin
rauwolfia serpentina
reserpine
sacubitril
spironolactone
terazosin
torsemide
triamterene</td></tr>
<tr><td><u>Thiazide diuretics</u>
bendroflumethiazide
chlorothiazide
chlorthalidone
hydrochlorothiazide
hydroflumethiazide
indapamide
methyclothiazide
polythiazide
trichlormethiazide</td><td><u>Beta blockers</u>
acebutolol
atenolol
betaxolol
bisoprolol
carteolol
carvedilol
labetalol
metoprolol
nadolol
nebivolol
penbutolol
pindolol
propranolol
sotalol
timolol</td></tr>
<tr><td><u>Angiotensin II receptor blockers (ARBs)</u>
azilsartan
candesartan
eprosartan
irbesartan
losartan
olmesartan
telmisartan
valsartan</td><td><u>Non-dihydropyridine calcium channel blockers</u>

diltiazem
verapamil
bepridil</td></tr>
</table>

© 2026 Gray SL et al. *JAMA Network Open.*

eTable 2. Description of Outcomes

	**Indicator of**	**Definition of high pathology[a]**
**AD-related**		
Thal phase	Aβ plaque distribution	3-5 vs. 0-2
Braak stage	Neurofibrillary tangle distribution	V or VI vs. 0 – IV
CERAD	Cortical neuritic plaque density	Moderate or frequent vs. none or sparse
ADNC	Continuum of neuropathologic changes that underlie AD (combined Thal phase, Braak stage and CERAD score)	intermediate or high vs. low or none
Cerebral amyloid angiopathy	Aβ accumulation in cerebral blood vessels	Mild, moderate, severe vs. none
**Vascular brain injury**		
Macroscopic infarcts[e]	Cerebrovascular disease	any vs. none
Cerebral microinfarcts	Cerebrovascular disease	any vs. none
Atherosclerosis	Large vessel intracranial vascular disease	moderate or severe vs. none or mild
Arteriolosclerosis	Small vessel intracranial vascular disease	moderate or severe vs. none or mild
**Other**		
LATE	Neurodegenerative disorder defined by aggregates of phosphorylated TDP-43 protein in the brain, especially the limbic system.	2-3 vs. 0-1
Lewy bodies	Presence of α-synuclein-positive Lewy bodies in specific brain region	Neocortex vs limbic, brainstem, or none
**Exploratory**		
$A\beta_{42}$ pg/microgram of protein	Quantity of amyloidogenic $A\beta_{42}$ in tissue extracts from 4 separate brain regions: frontal, occipital, temporal and parietal	higher number indicates greater amyloid accumulation
Tau phosphorylation burden (% positive tissue)	Percent area of positive immunohistochemical staining for hyper-phosphorylated Tau protein in digitized histology slides of frontal, occipital, temporal, parietal, and hippocampal brain regions. Additional regions examined that aligned with Braak I-IV staging (CA1, Entorhinal cortex, Subiculum, transentorhinal cortex)	higher percentage indicates greater pathologic tau burden

Abbreviations: AD, Alzheimer disease; ADNC Alzheimer's disease neuropathologic change; CA1: hippocampal Cornu ammonis subfield 1; CERAD, Consortium to Establish a Registry for Alzheimer's Disease; LATE limbic-predominant age-related TDP-43 encephalopathy;

[a]Higher neuropathology category(ies) shown first

© 2026 Gray SL et al. *JAMA Network Open.*

eTable 3. Sources of Exposure and Covariate Data

Data type	ACT study visit	Chart review	Pharmacy dispensing data or electronic health record
Exposure			
Antihypertensive exposure		pre-1977	post-1977
Demographics			
ACT study cohort[a,b]	X		
Age at last ACT visit[b]	X		
Age at first known antihypertensive use[a]		X	X
Age at death[a]	X		
Sex[a,b]	X		
Self-reported race and ethnicity	X		
Education[b]	X		
Self-reported health	X		
Self-reported exercise	X		
Biospecimen and Blood pressure			
APOE genotype[b]	X		
Systolic blood pressure[a]	X	X	X
Diastolic blood pressure[a]	X	X	X
Comorbidities			
Atrial fibrillation[a,b]		X	**ICD-9**: 427.31, 427.32,<br>**ICD-10**: I48.x
Diabetes[a.b]	X	X	**ICD-9**: 250.xx<br>**ICD-10**: E10.xxx, E11.xxx, E12.xxx, E13.xxx
Stroke[a,b]	X	X	**ICD-9**: 431.x, 434.x, 438.x<br>**ICD-10**: 160.9, I63.xx, 169.3xx
Myocardial infarction[a]	X	X	**ICD-9**: 410.x<br>**ICD-10**: I21.xx
Depressive symptoms	CES-D score ≥ 10		**ICD-9**: 296.2x, 296.3x, 311<br>**ICD10**: F32.xx, F33.xx
Coronary artery disease[b]	X		**ICD-9**:<br>Myocardial infarction: 410.x<br>Angina: 411.1, 411.81, 411.89, 413.0, 413.1, 413.9<br>CABG: V45.81<br>Angioplasty: V45.82<br>**ICD-10**:<br>Myocardial infarction: I21.xx<br>Angina: I20.0, I20.1, I20.8, I20.9, I24.0, I24.8, I24.9<br>CABG: Z95.1<br>Angioplasty: Z95.5, Z98.61
Heart failure[a,b]	X	X	**ICD-9:** 398.91, 402.11, 402.91, 404.11, 404.13, 404.93, 428.0, 428.1, 428.2x,428.3x,428.4x,428.9<br>**ICD-10:** I09.81, I11.0, I30.0, I13.2, I50.2x, I50.3x, I50.4x, I50.9, I50.1
Dementia[b] and AD	X		

Abbreviations: ACT, Adult Changes in Thought; AD, Alzheimer disease; APOE, Apolipoprotein; CES-D, Center of Epidemiologic Studies Depression; ICD, International Classification of Diseases
[a]Covariates in outcome models; [b]Covariates in selection models.

© 2026 Gray SL et al. *JAMA Network Open.*

eTable 4. Characteristics of ACT Autopsy Sample by Long-Term Angiotensin II Antihypertensive Exposures[a,b]

Characteristic	Angiotensin II-stimulating		Angiotensin II-inhibiting	
	<15 years N=574	≥ 15 years N=182	<15 years N=484	≥15 years N=272
	N (%)	N (%)	N (%)	N (%)
**ACT study entry**				
ACT study cohort				
Original	332 (57.8)	96 (52.7)	286 (59.1)	142 (52.2)
Expansion	126 (22.0)	45 (24.7)	111 (22.9)	60 (22.1)
Replacement	116 (20.2)	41 (22.5)	87 (18.0)	70 (25.7)
Age at index in years, mean (SD)	70.6 (13.0)	55.8 (11.3)	71.1 (14.0)	59.8 (11.2)
Age at death in years, mean (SD)	89 (6.6)	90 (5.8)	89.1 (6.6)	89.4 (6.0)
Time from first angiotensin II antihypertensive medication to death, mean (SD), y	18.4 (12.2)	34.2 (10.1)	18 (13.3)	29.6 (10.3)
Sex				
Female	317 (55.2)	123 (67.6)	289 (59.7)	151 (55.5)
Male	257 (44.8)	59 (32.4)	195 (40.3)	121 (44.5)
Race and ethnicity[c]				
Asian	8 (1.4)	8 (4.4)	6 (1.2)	10 (3.7)
Other	18 (3.1)	11 (6.0)	17 (3.5)	12 (4.4)
White	547 (95.3)	163 (89.6)	460 (95.0)	250 (91.9)
Some college education	426 (74.2)	125 (68.7)	347 (71.7)	204 (75.0)
Fair to poor self-rated health	150 (26.1)	50 (27.5)	124 (25.6)	76 (27.9)
Regular exercise[d]	221 (38.5)	62 (34.1)	194 (40.1)	89 (32.7)
APOE ε4	150 (26.1)	53 (29.1)	133 (27.5)	70 (25.7)
**Summarized over study follow-up (at death)**				
Blood pressure				
Annual systolic blood pressure, mean (SD), mm Hg[e]	133.9 (12.8)	141.6 (9.7)	134.6 (12.7)	137.9 (12.0)
Annual diastolic blood pressure, mean (SD), mm Hg[e]	72.5 (6.9)	77.9 (6.2)	72.7 (7.2)	75.6 (6.7)
No. of years with uncontrolled blood pressure, mean (SD)[f]	4.3 (4.6)	14.3 (7.5)	4.4 (4.9)	10.9 (8.0)
Antihypertensive medication				
Angiotensin II-stimulating antihypertensive medication				
Ever used	402 (70.0)	182 (100.0)	354 (73.1)	230 (84.6)
Total person-years of exposure, mean (SD)	4.6 (4.6)	24.2 (7.5)	6.9 (8.3)	13.5 (11.3)
Angiotensin II-inhibiting antihypertensive medication				
Ever used	534 (93.0)	172 (94.5)	434 (89.7)	272 (100.0)

© 2026 Gray SL et al. *JAMA Network Open.*

eTable 4. Characteristics of ACT Autopsy Sample by Long-Term Angiotensin II Antihypertensive Exposures, continued[a,b]

Total person-years of exposure, mean (SD)	10.4 (7.9)	17.6 (10.6)	6.4 (4.5)	22.4 (6.1)
Any use of other antihypertensive medication	392 (68.3)	159 (87.4)	326 (67.4)	225 (82.7)
History of comorbidities at death				
Diabetes	181 (31.5)	64 (35.2)	135 (27.9)	110 (40.4)
Stroke	319 (55.6)	110 (60.4)	265 (54.8)	164 (60.3)
Myocardial infarction	318 (55.4)	99 (54.4)	241 (49.8)	176 (64.7)
Atrial fibrillation	328 (57.1)	101 (55.5)	247 (51.0)	182 (66.9)
Heart failure	376 (60.1)	131 (72.0)	310 (64.0)	197 (72.4)
Coronary artery disease	349 (60.8)	107 (58.8)	255 (52.7)	201 (73.9)
High depressive symptoms[g]	345 (60.1)	118 (64.8)	287 (59.3)	176 (64.7)
Dementia[h]	272 (47.4)	82 (45.1)	233 (48.1)	121 (44.5)
Alzheimer disease[h]	227 (39.5)	65 (35.7)	194 (40.1)	98 (36.0)

Abbreviations: ACT, Adult Changes in Thought

[a]Results are presented as number and percent unless specified.

[b]Exposure groups are not mutually exclusive. Participants could be long-term users of both stimulating and inhibiting agents.

[c]Other race and ethnicity included American Indian or Alaska Native, Black, Hispanic ethnicity, and other races or multiple races.

[d]Performing one of several listed activities for 15 minutes or more, 3 times or more per week.

[e]Mean annual systolic and diastolic blood pressure defined as each individual's mean of annual systolic and diastolic blood pressure across the years with angiotensin II antihypertensive medication exposure.

[f]Uncontrolled blood pressure defined as systolic blood pressure of 140 mm Hg or higher and/or diastolic blood pressure of 90 mm Hg or higher.

[g]Center for Epidemiologic Studies Depression scale of 10 or more or *International Classification of Diseases* diagnosis

[h]Participants who screened positive for cognitive impairment during an ACT study visit underwent a standardized diagnostic evaluation for dementia by a multidisciplinary consensus conference and standard criteria were used for diagnoses of dementia and Alzheimer disease.

© 2026 Gray SL et al. *JAMA Network Open.*

eTable 5. Person-Years (PYs) of Exposure According to Antihypertensive Class

	PYs	% total PYs	N (%) of people with exposure ≥15 years
**Angiotensin II-stimulating antihypertensives**			
Angiotensin II receptor blocker	821.8	10.4	7 (0.9%)
Dihydropyridine calcium channel blocker	1677.0	21.2	21 (2.8%)
Thiazide diuretic	5417.0	68.4	136 (18.0%)
**Angiotensin II-inhibiting antihypertensives**			
Angiotensin-converting enzyme inhibitor	3791.8	33.0	70 (9.3%)
Beta blocker[a]	6199.8	53.9	159 (21.0%)
Non-dihydropyridine calcium channel blocker	1505.3	13.1	30 (4.0%)

[a] 2183.8 person-years of beta-blocker monotherapy.

© 2026 Gray SL et al. *JAMA Network Open.*

eTable 6. Distribution of Neuropathology Outcomes[a]

	Sample	N (%)
**Alzheimer's disease-related**		
Aβ plaque distribution (Thal phase)	748	
0		92 (12.3)
1		87 (11.6)
2		68 (9.1)
3		141 (18.9)
4		*213 (28.5)*
5		*147 (19.7)*
Neurofibrillary tangle distribution (Braak stage)	750	
0		20 (2.7)
I		51 (6.8)
II		116 (15.5)
III		125 (16.7)
IV		*150 (20.0)*
V		*191 (25.5)*
Cortical neuritic plaque density (CERAD)	756	
None		176 (23.3)
Sparse		167 (22.1)
Moderate		200 (26.5)
Frequent		*213 (28.2)*
ADNC	742	
None		89 (12.0)
Low		222 (29.9)
Intermediate		*223 (30.1)*
High		*208 (28.0)*
Cerebral amyloid angiopathy	756	
None		369 (48.8)
Mild		*143 (18.9)*
Moderate		*227 (30.0)*
Severe		*17 (2.2)*
**Vascular brain injury**		
Macroscopic infarcts (any)	753	268 (35.6)
Cerebral microinfarcts (any)	753	377 (50.1)
Atherosclerosis level	745	
None		32 (4.3)
Mild		159 (21.3)
Moderate		*489 (65.6)*
Severe		*65 (8.7)*
Arteriolosclerosis level	661	
None		6 (0.9)
Mild		147 (22.2)
Moderate		*335 (50.7)*
Severe		*173 (26.2)*
**Other**		
LATE stages	736	
0		370 (50.3)
1		159 (21.6)
2		*192 (26.1)*
3		*15 (2.0)*

© 2026 Gray SL et al. *JAMA Network Open.*

eTable 6. Distribution of Neuropathology Outcomes,[a] continued

	Sample	N (%)
Presence of Lewy bodies	755	
None		587 (77.7)
Brainstem		26 (3.4)
Limbic		78 (10.3)
Neocortex		*64 (8.5)*

Abbreviations: ADNC Alzheimer disease neuropathologic change; CERAD, Consortium to Establish a Registry for Alzheimer Disease; LATE limbic-predominant age-related TDP-43 encephalopathy.
[a]Definition of high levels of pathology are in italics.

© 2026 Gray SL et al. *JAMA Network Open.*

eTable 7. Characteristics of ACT Autopsy Sample by Neuropathology Outcome Status

High level of neuropathology burden	Outcome prevalence[a]	PYs of angiotensin II stimulating	PYs of angiotensin II inhibiting	Atrial fibrillation	Diabetes	Myocardial infarction	Stroke	Heart failure
	N (%)	Mean (SD)	Mean (SD)	N (%)	N (%)	N (%)	N (%)	N (%)
**Alzheimer disease related**								
Thal phase								
Yes (Phase 3-5)	501 (67.0)	9.7 (10.0)	12.4 (9.2)	276 (55.1)	155 (30.9)	283 (56.5)	275 (54.9)	317 (63.3)
No (Phase 1 and 2)	247 (33.0)	8.8 (10.1)	12.0 (9.3)	148 (60.0)	85 (34.4)	129 (52.2)	148 (59.9)	185 (74.9)
Braak stage								
Yes (V and VI)	288 (38.4)	9.8 (10.1)	12.8 (9.5)	148 (51.4)	82 (28.5)	160 (55.6)	160 (55.6)	172 (59.7)
No (I-IV)	462 (61.6)	8.9 (9.9)	11.7 (9.0)	277 (60.0)	161 (34.9)	254 (55.0)	265 (57.4)	333 (72.1)
CERAD								
Yes (mod/frequent)	413 (54.6)	9.7 (10.1)	12.7 (9.4)	223 (54.0)	136 (32.9)	234 (56.7)	232 (56.2)	256 (62.0)
No	343 (45.4)	8.8 (9.8)	11.5 (8.9)	206 (60.1)	109 (31.8)	183 (53.4)	197 (57.4)	251 (73.2)
ADNC								
Yes (intermed/high)	431 (58.1)	9.5 (10.0)	12.4 (9.2)	234 (54.3)	130 (30.2)	242 (56.2)	235 (54.5)	265 (61.5)
No	311 (41.9)	9.1 (10.1)	11.9 (9.3)	186 (59.8)	108 (34.7)	167 (53.7)	184 (59.2)	235 (75.6)
Cerebral amyloid angiopathy								
Yes (any)	387 (51.2)	9.0 (10.2)	12.1 (9.1)	213 (55.0)	114 (29.5)	205 (53.0)	220 (56.9)	245 (63.3)
No	369 (48.8)	9.6 (9.8)	12.3 (9.3)	216 (58.5)	131 (35.5)	212 (57.5)	209 (56.6)	262 (71.0)
**Vascular brain injury**								
Macroscopic infarcts								
Yes (any)	268 (35.6)	10.4 (9.9)	13.1 (9.3)	160 (59.7)	90 (33.6)	147 (54.9)	198 (73.9)	184 (68.7)
No	485 (64.4)	8.6 (9.9)	11.7 (9.1)	268 (55.3)	153 (31.8)	268 (55.3)	229 (47.2)	322 (66.4)
Cerebral microinfarcts								
Yes (any)	377 (50.1)	9.2 (9.9)	12.2 (9.0)	224 (59.4)	132 (35.0)	213 (56.5)	244 (64.7)	268 (71.1)
No	376 (49.9)	9.4 (10.2)	12.2 (9.4)	204 (54.3)	111 (29.5)	203 (54.0)	183 (48.7)	237 (63.0)

© 2026 Gray SL et al. *JAMA Network Open.*

eTable 7. Characteristics of ACT Autopsy Sample by Neuropathology Outcome Status, continued

High level of neuropathology burden	Outcome prevalence[b]	PYs of angiotensin II stimulating	PYs of angiotensin II inhibiting	Atrial fibrillation	Diabetes	Myocardial infarction	Stroke	Heart failure
	N (%)	Mean (SD)	Mean (SD)	N (%)	N (%)	N (%)	N (%)	N (%)
Atherosclerosis								
Yes (mod/severe)	554 (74.4)	9.7 (10.1)	12.4 (9.3)	316 (57.0)	184 (33.2)	302 (54.5)	327 (59.0)	371 (67.0)
No	191 (25.6)	8.4 (9.7)	11.7 (9.1)	105 (55.0)	59 (30.9)	106 (55.5)	97 (50.8)	126 (66.0)
Arteriolosclerosis								
Yes (mod/severe)	508 (76.9)	9.7 (10.2)	13.1 (9.5)	287 (56.5)	174 (34.3)	285 (56.1)	313 (61.6)	336 (66.1)
No	153 (23.1)	8.4 (10.0)	10.7 (8.3)	94 (61.4)	47 (30.7)	76 (49.7)	63 (41.2)	102 (66.7)
**Other**								
LATE-stage present								
Yes (2 or 3)	207 (28.1)	8.9 (9.8)	12.7 (9.8)	107 (51.7)	48 (23.2)	113 (54.6)	109 (52.7)	129 (62.3)
No	529 (71.9)	9.6 (10.1)	12.1 (9.0)	313 (59.2)	189 (35.7)	291 (55.0)	306 (57.8)	365 (69.0)
Presence of Lewy bodies in neocortex								
Yes (any)	64 (8.5)	8.8 (9.5)	13.0 (9.5)	31 (48.4)	14 (21.9)	33 (51.6)	44 (68.8)	34 (53.1)
No	691 (91.5)	9.3 (10.1)	12.1 (9.2)	398 (57.6)	230 (33.3)	384 (55.6)	385 (55.7)	472 (68.3)

Abbreviations: ADNC, Alzheimer disease neuropathologic change; CERAD, Consortium to Establish a Registry for Alzheimer Disease; LATE, limbic-predominant age-related TDP-43 encephalopathy; SD Standard deviation

[b]Not all 756 participants had all the neuropathology outcomes.

© 2026 Gray SL et al. *JAMA Network Open.*

eFigure 1. Activity of Antihypertensive Sub-Classes in the Renin-Angiotensin System

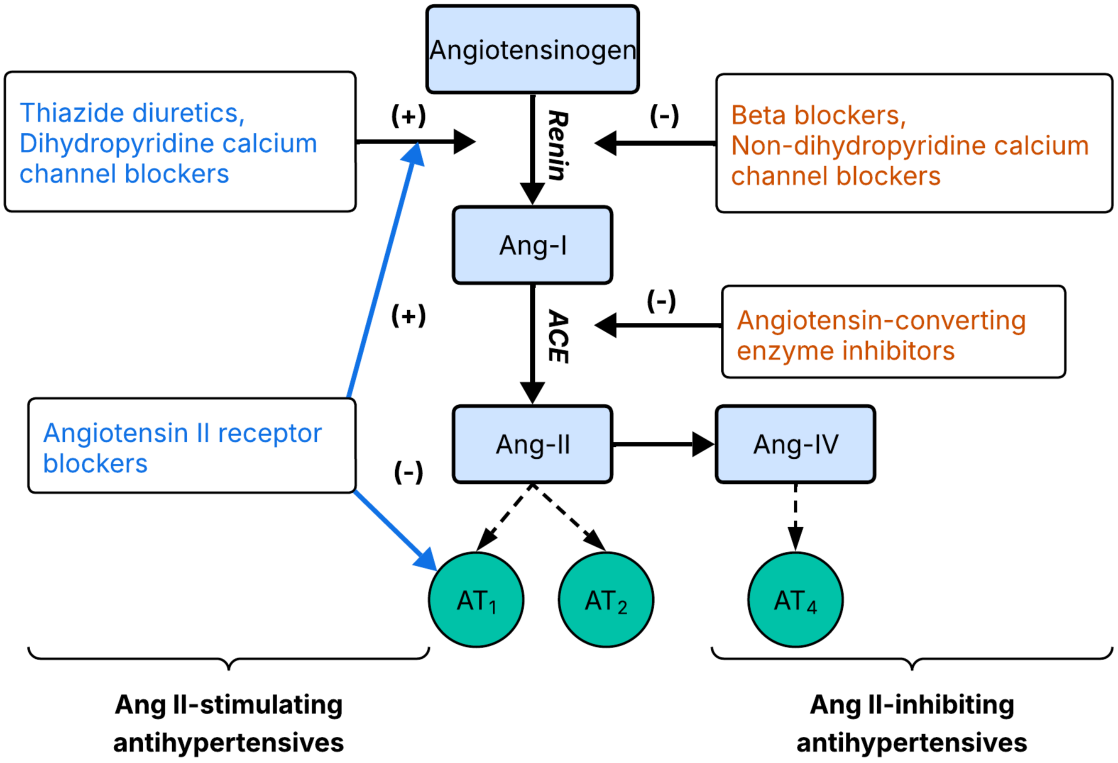

Thiazides and dihydropyridine calcium channel blockers increase renin. β–blockers reduce β1-mediated renin production. Renin generates angiotensin I (Ang-I), which is converted into angiotensin II (Ang II) by angiotensin-converting enzyme (ACE), which exerts physiological effects by binding to AT_1 or AT_2 or may be further metabolized into Ang-IV, which binds to AT4. ACE inhibitors directly inhibit ACE activity, thereby inhibiting angiotensin II production. Angiotensin receptor 1 blockers (ARBs) inhibit angiotensin II activity directly at the AT_1 receptor but leave angiotensin II production intact.

ACE reportedly degrades β-amyloid (Aβ), a major component of the cerebral neuritic plaques associated with Alzheimer disease. ACE inhibitors may inhibit this degradation, thus facilitating Aβ plaque accumulation. ARBs selectively inhibit Ang II at angiotensin receptor 1 (AT_1) without inhibiting ACE, allowing ACE to degrade Aβ. Moreover, Ang II and Ang IV activity have been associated with protection from ischemia via activity at AT_2 and possibly AT_4. In addition, Ang II and Ang IV activity have been associated with direct effects on memory. Taken together, antihypertensives that increase activity at AT_2 and AT_4 (Ang II-stimulating antihypertensives) are hypothesized to have greater brain protective effects than those that decrease activity at the same receptors (inhibiting antihypertensives). Recreated from reference 4.

© 2026 Gray SL et al. *JAMA Network Open.*

eFigure 2. Flow Diagram of Study Sample

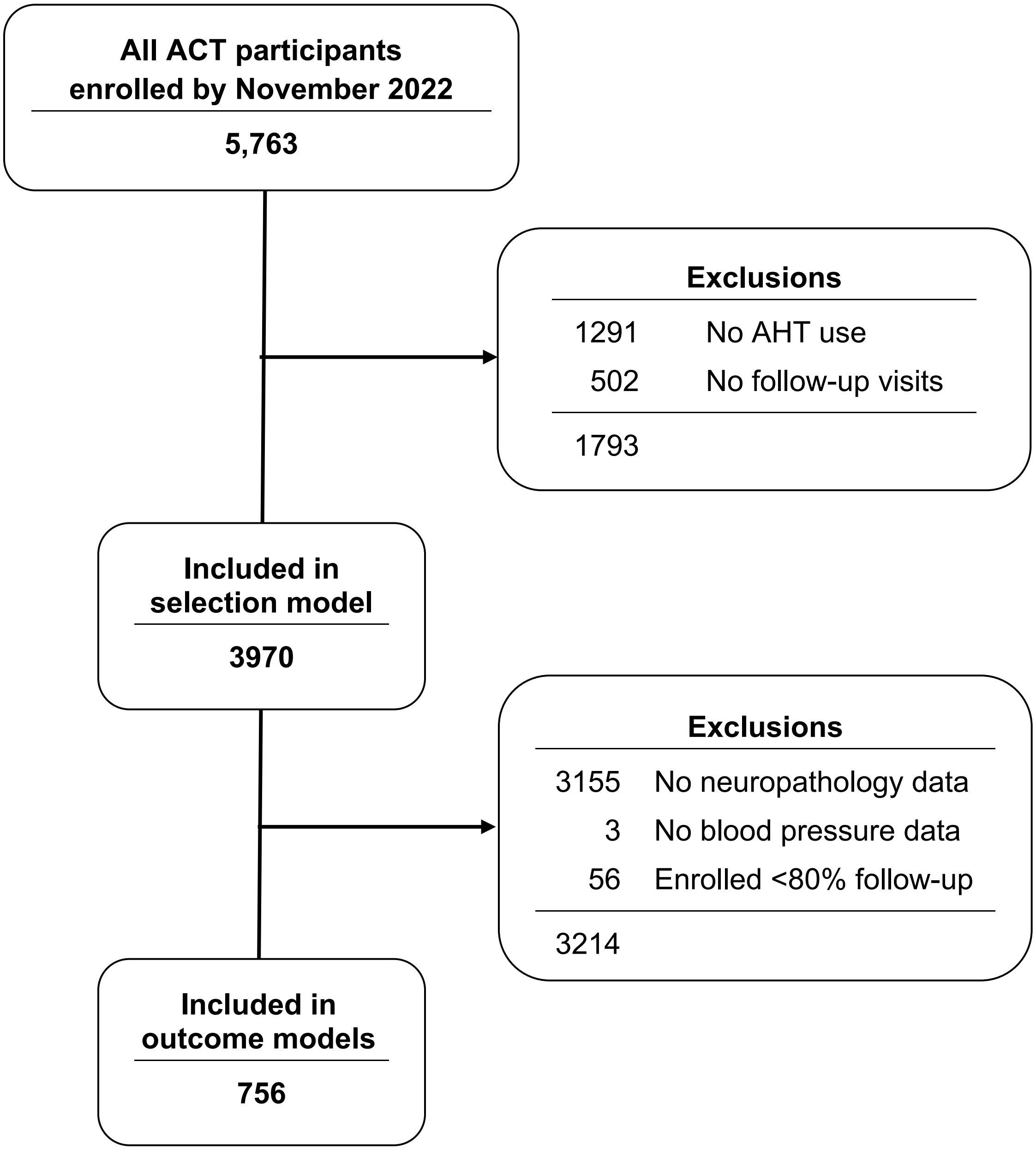

© 2026 Gray SL et al. *JAMA Network Open.*

eFigure 3. Person-Years (PYs) of Exposure by Calendar Year According to Type of Angiotensin II Exposure

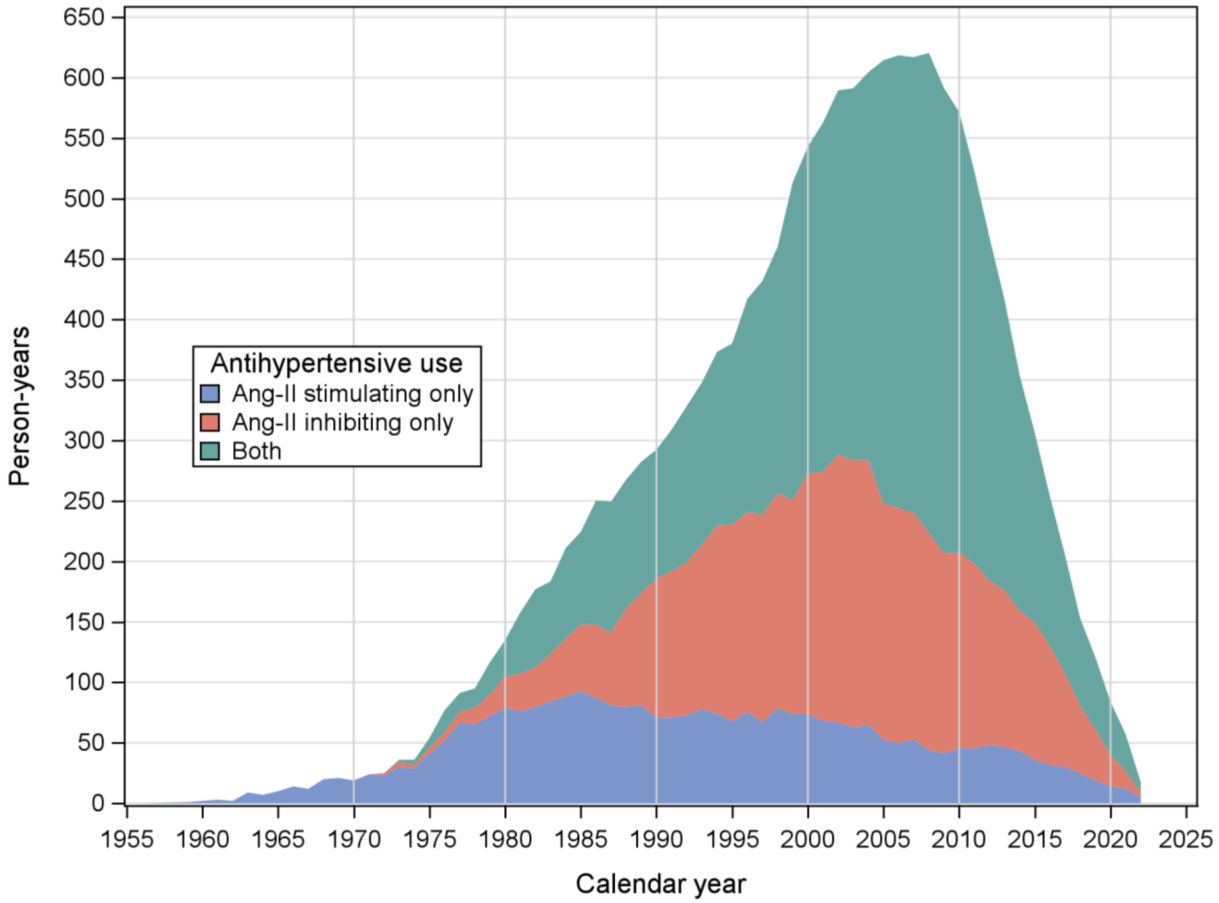

This cohort study used data from the autopsy sample from the Adult Changes in Thought (ACT) study with enrollment from February 24,1994 to November 25, 2022. Medication exposures were available from chart review and computerized pharmacy data prior to ACT enrollment. This figure illustrates the total person year of exposure for all participants during study follow-up, from index date to death, according to pattern of use: angiotensin II-stimulating only, angiotensin II-inhibiting only or users of both types of angiotensin II antihypertensives. Angiotensin II-stimulating antihypertensives included angiotensin II receptor blockers, dihydropyridine calcium channel blockers, and thiazides. Angiotensin II-inhibiting antihypertensives included angiotensin-converting enzyme inhibitors, beta-blockers, and non-dihydropyridine calcium channel blockers.

© 2026 Gray SL et al. *JAMA Network Open.*

eFigure 4. Person-Years (PYs) of Exposure by Calendar Year According to Angiotensin II Antihypertensive Subclass

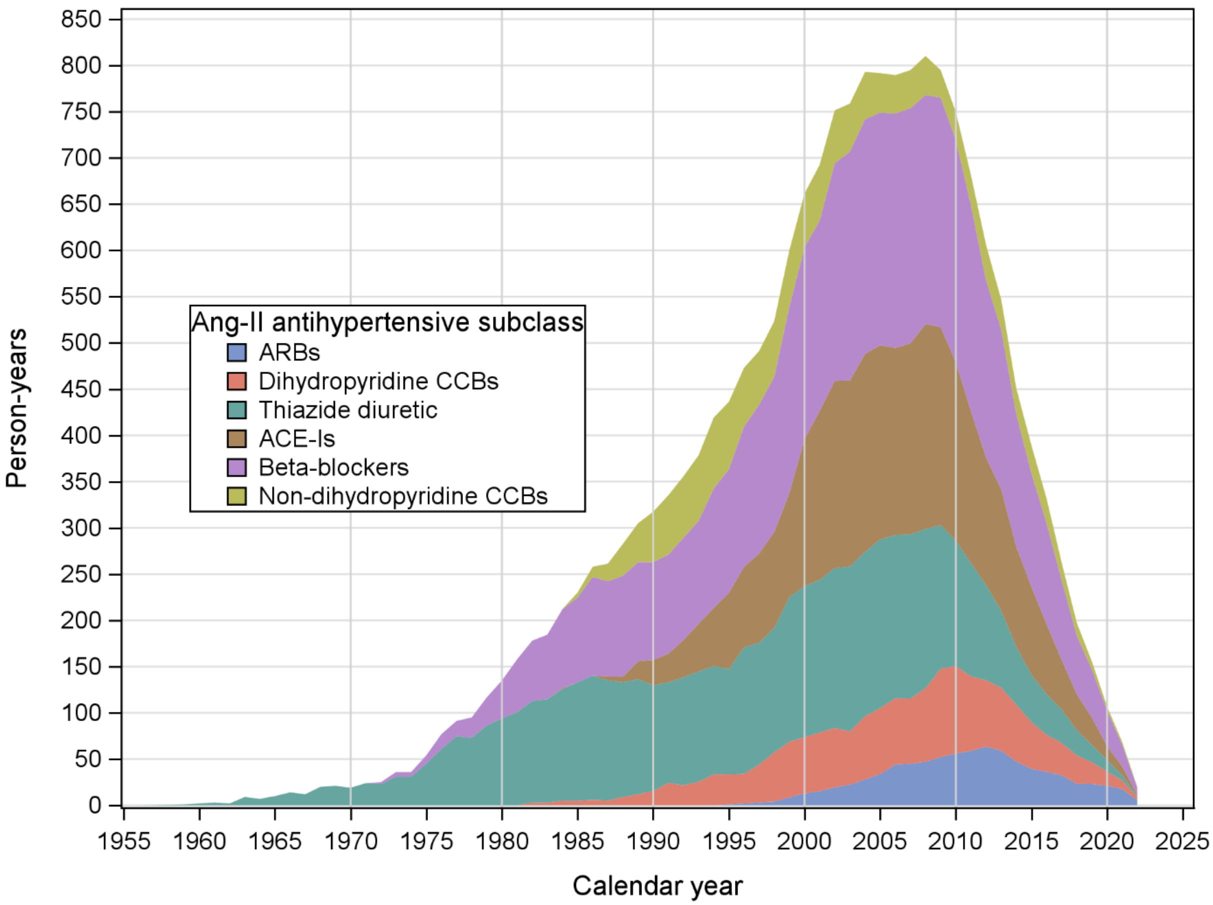

This cohort study used data from the autopsy sample from the Adult Changes in Thought (ACT) study with enrollment from February 24, 1994 to November 25, 2022. Medication exposures were available from chart review and computerized pharmacy data prior to ACT enrollment. This figure illustrates the total person year of exposure for all participants during study follow-up, from index date to death, according to subclass of angiotensin II-stimulating medication. Angiotensin II-stimulating antihypertensives included angiotensin II receptor blockers, dihydropyridine calcium channel blockers, and thiazides. Angiotensin II-inhibiting antihypertensives included angiotensin-converting enzyme inhibitors, beta-blockers, non-dihydropyridine calcium channel blockers.

© 2026 Gray SL et al. *JAMA Network Open.*

eFigure 5. Associations Between Cumulative Person-Years (PYs) of Antihypertensive Exposure and Neuropathology Outcomes Not Adjusting for Blood Pressure

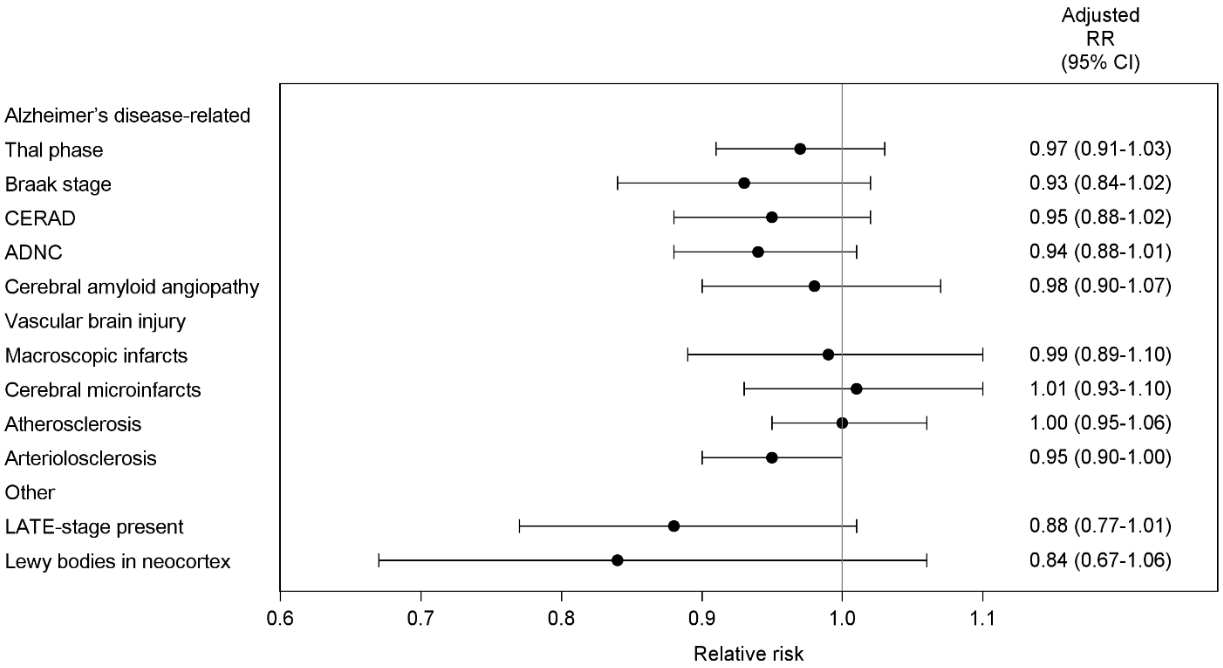

Relative risk (RR) estimated from modified Poisson regression for each neuropathology outcome comparing 5 additional PYs of angiotensin II–stimulating with 5 additional PYs of angiotensin II–inhibiting antihypertensive medication exposure. High level of neuropathology, defined as Thal phase (3-5), Braak stage (V or VI), Consortium to Establish a Registry for Alzheimer Disease (CERAD) level (moderate or frequent), Alzheimer disease neuropathologic change (ADNC) score (intermediate or high), cerebral amyloid angiopathy (any), macroscopic infarcts (any), cerebral infarcts (any), atherosclerosis (moderate or severe), arteriolosclerosis (moderate or severe), and limbic-predominant age-related TDP-43 encephalopathy (LATE) present (2-3). Models for macroscopic infarcts did not adjust for stroke because of collinearity expected between these 2 variables. Lewy bodies in neocortex results are presented as odds ratios from ordinal logistic regression (the risk of presence of Lewy bodies in the neocortical region vs in the limbic or brainstem regions or absence of Lewy bodies). Models adjusted for age at death, age at first known antihypertensive medication use, sex, Adult Changes in Thought cohort, use of other antihypertensive medications, and history of atrial fibrillation, diabetes, myocardial infarction, stroke, and heart failure any time prior to death.

© 2026 Gray SL et al. *JAMA Network Open.*

eFigure 6. Associations Between Cumulative Person-Years (PYs) of Antihypertensive Exposure and Neuropathology Outcomes Adjusting for Uncontrolled Blood Pressure

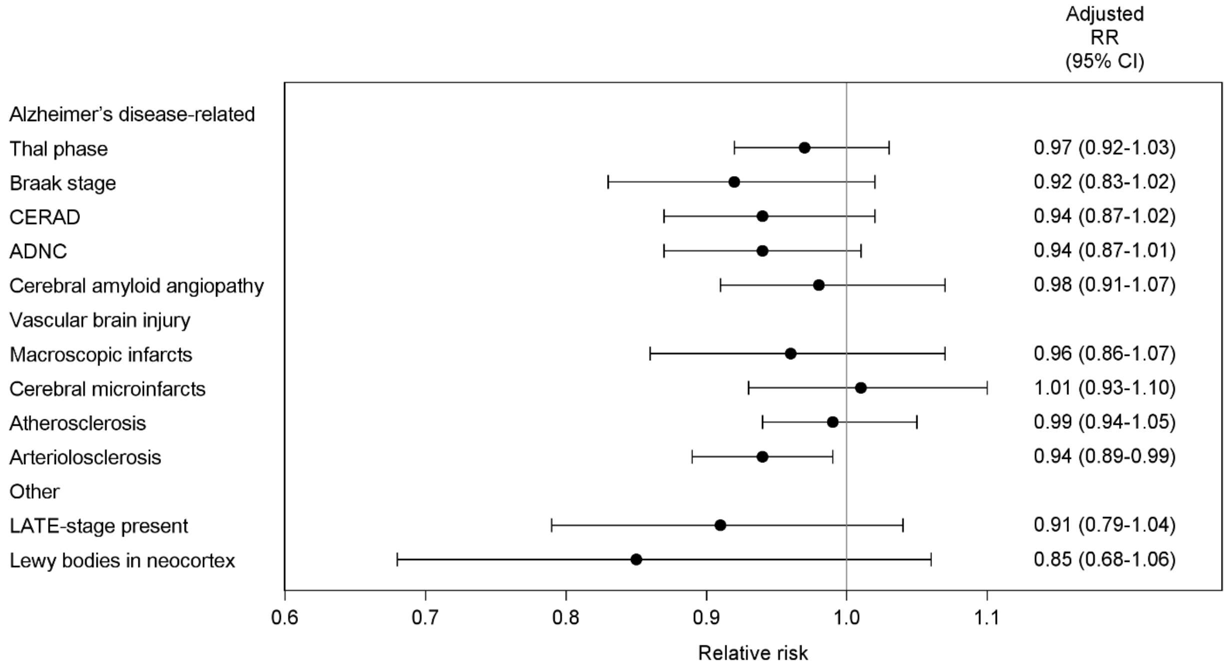

Relative risk (RR) estimated from modified Poisson regression for each neuropathology outcome comparing 5 additional PYs of angiotensin II–stimulating with 5 additional PYs of angiotensin II–inhibiting antihypertensive medication exposure. High level of neuropathology, defined as Thal phase (3-5), Braak stage (V or VI), Consortium to Establish a Registry for Alzheimer Disease (CERAD) level (moderate or frequent), Alzheimer disease neuropathologic change (ADNC) score (intermediate or high), cerebral amyloid angiopathy (any), macroscopic infarcts (any), cerebral infarcts (any), atherosclerosis (moderate or severe), arteriolosclerosis (moderate or severe), and limbic-predominant age-related TDP-43 encephalopathy (LATE) present (2-3). Models for macroscopic infarcts did not adjust for stroke because of collinearity expected between these 2 variables. Lewy bodies in neocortex results are presented as odds ratios from ordinal logistic regression (the risk of presence of Lewy bodies in the neocortical region vs in the limbic or brainstem regions or absence of Lewy bodies). Models adjusted for age at death, age at first known antihypertensive medication use, sex, Adult Changes in Thought cohort, proportion of total person-year with blood pressure available that the mean systolic blood pressure was 140 mm Hg or higher and/or diastolic blood pressure of 90 mm Hg or higher as a measure of amount of time with uncontrolled blood pressure, use of other antihypertensive medications, and history of atrial fibrillation, diabetes, myocardial infarction, stroke, and heart failure any time prior to death.

© 2026 Gray SL et al. *JAMA Network Open.*

eFigure 7. Associations Between Cumulative Person-Years (PYs) of Antihypertensive Exposure and Neuropathology Outcomes in New Users of Antihypertensives[a]

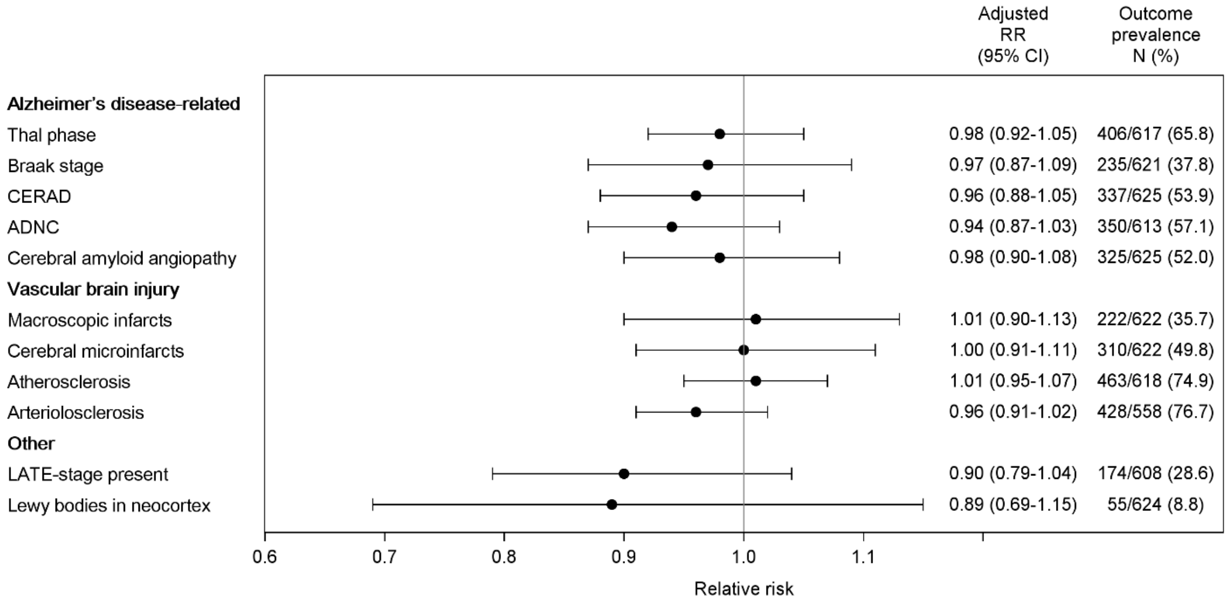

Relative risk (RR) estimated from modified Poisson regression for each neuropathology outcome comparing 5 additional PYs of angiotensin II–stimulating with 5 additional PYs of angiotensin II–inhibiting antihypertensive medication exposure. High level of neuropathology, defined as Thal phase (3-5), Braak stage (V or VI), Consortium to Establish a Registry for Alzheimer Disease (CERAD) level (moderate or frequent), Alzheimer disease neuropathologic change (ADNC) score (intermediate or high), cerebral amyloid angiopathy (any), macroscopic infarcts (any), cerebral infarcts (any), atherosclerosis (moderate or severe), arteriolosclerosis (moderate or severe), and limbic-predominant age-related TDP-43 encephalopathy (LATE) present (2-3). Models for macroscopic infarcts did not adjust for stroke because of collinearity expected between these 2 variables. Lewy bodies in neocortex results are presented as odds ratios from ordinal logistic regression (the risk of presence of Lewy bodies in the neocortical region vs in the limbic or brainstem regions or absence of Lewy bodies). Models adjusted for age at death, age at first known antihypertensive medication use, sex, Adult Changes in Thought cohort, mean annual diastolic and systolic blood pressure across the years with angiotensin II antihypertensive medication exposure, use of other antihypertensive medications, and history of atrial fibrillation, diabetes, myocardial infarction, stroke, and heart failure any time prior to death.

© 2026 Gray SL et al. *JAMA Network Open.*
