# Supplementary material for: Angiotensin II–Stimulating Antihypertensive Medications and Dementia-Related Neuropathology
Source: JAMA Netw Open. 2026 Feb 11;9(2):e2559113. doi: 10.1001/jamanetworkopen.2025.59113 (PMC12895290; doi:10.1001/jamanetworkopen.2025.59113)
Supplement: Supplement 2. — Data Sharing Statement [file jamanetwopen-e2559113-s002.pdf]

Data Sharing Statement

Gray. Angiotensin II–Stimulating Antihypertensive Medications and Dementia-Related Neuropathology. *JAMA Netw Open*. Published February 11, 2026.
doi:10.1001/jamanetworkopen.2025.59113

Data
Data available: No

Additional Information
Explanation for why data not available: Data from this analysis cannot be made publicly available for ethical and legal reasons. In order to replicate our findings, a researcher may need access to personal health identifiers (PHI) Version 8/6/2024 2 including dates of birth and death, dates of diagnoses, and ages over 89. These are required variables for the analysis, and we cannot publicly release this information without IRB approval and a Data Use Agreement with interested researchers. However, the datasets used and/or analyzed in the current study are available upon reasonable request and execution of appropriate human subjects review and data sharing agreements by following the process described on the Adult Changes in Thought (ACT) website: actagingresearch.org.
